# Tuberculosis and One Health – What is in a Name?

**DOI:** 10.3389/fvets.2015.00054

**Published:** 2015-11-11

**Authors:** Susanna Sternberg Lewerin

**Affiliations:** ^1^Department of Biomedical Sciences and Veterinary Public Health, Swedish University of Agricultural Sciences (SLU), Uppsala, Sweden

**Keywords:** zoonotic, tuberculosis, epidemiology, nomenclature, terminology

## Introduction

Most control programs for bovine tuberculosis include reporting to both veterinary and public health authorities, and measures to prevent transmission from animals to humans. On the other hand, reporting of human cases to veterinary authorities is rare, unless an animal source is suspected. Exchange of data and strategic discussions between veterinary and public health authorities would strengthen tuberculosis surveillance in both animal and human populations.

A One Health approach is clearly warranted for tuberculosis. The disease has similarly serious consequences for humans and a broad range of animal species, and it has been strongly advocated as a One Health issue (1). Yet, tuberculosis cases in humans and animals are commonly treated as separate problems (2, 3).

Tuberculosis is caused by bacteria within the *Mycobacterium tuberculosis* complex (MTBC). “Bovine tuberculosis” usually means infection in cattle with *Mycobacterium bovis* (3, 4). However, this definition may be too restrictive, and the term bovine tuberculosis has been proposed to signify infection in cattle with any bacteria in the MTBC (5, 6). Similarly, “human tuberculosis” usually refers to infection in humans with *M. tuberculosis*, while “zoonotic tuberculosis” refers to human infection with *M*. bovis (7). However, for control and monitoring purposes, as well as a One Health approach, it would be preferable to use the terms “human tuberculosis” and “bovine tuberculosis” for infection with all bacteria within the MTBC in humans and cattle, respectively. Consequently, infections in other host species could be named after the host species, complemented by specifying the infecting bacterial species. In the following, the term “tuberculosis” is used for infection with bacteria within the MTBC, regardless of host species.

## Tuberculosis in Different Host Populations

Bacteria within the MTBC have a broad host range, although there are differences in host susceptibility as well as in the detailed pathophysiology of the infection (4, 8, 9). Factors such as host species, individual immunity, route of infection, and infectious dose have a strong impact on the course of the disease, regardless of bacterial species (8, 10), and it may be difficult to discern the exact pathogenic peculiarities of each species.

Differences in the infectious dose for a certain host species via a certain route are reflected in the epidemiology of the disease in different populations (8, 10). In a high prevalence environment, the probability of exposure and infection is high for any susceptible host as seen in sporadic cases or outbreaks of tuberculosis in species, such as sheep, pigs, and horses.

Infection by *M. bovis* has been reported in a wide range of domesticated and wild animals. Although not as frequent a cause as *M. tuberculosis*, a substantial number of humans with tuberculosis are infected with *M. bovis*. The prevalence of *M. bovis* in human tuberculosis is generally higher in low-income regions with a high prevalence in cattle. However, it has been proposed that the global prevalence of *M. bovis* in humans is underestimated (11–15). In the absence of immunosuppression, human-to-human transmission of *M. bovis* is rare but has been observed (15, 16). *Mycobacterium caprae*, previously regarded as a caprine subtype of *M. bovis*, has also been identified as the causative agent of tuberculosis in domestic and wild animals, as well as humans.

*Mycobacterium tuberculosis* is most commonly isolated from people, but in regions with a high prevalence in the human population, the infection may spill over to animals. Most animal isolates of *M. tuberculosis* originate from cattle, pigs, dogs, and Indian elephants, but the infection has also been demonstrated in captive and free-living wildlife. Molecular epidemiology has demonstrated transmission between humans and animals, in both directions (17–20). Other MTBC species, such as *Mycobacterium microti*, *Mycobacterium pinnipedii*, and *Mycobacterium africanum*, have also been isolated from various host species. Genomic studies into bacterial evolution suggest that sea mammals, in addition to human dispersals, may have played a role in transmitting tuberculosis across the ocean in Pre-Columbian times, illustrating the complexity of the host–pathogen relationship (21).

## Epidemiology of Tuberculosis in Different Populations

Infectious dose and route of infection are important factors for the pathogenesis of the disease in individual hosts. Consequently, population dynamics, spatial distribution, contact patterns, and population sizes influence disease epidemiology. Efforts to categorize host species as “maintenance hosts” and “spillover hosts” are sometimes contradictory (8, 10, 22), probably due to the fact that the populations of these species differ between regions and ecological systems. The maintenance population may consist of one or several host species, depending on how the effective critical community size is achieved in different ecosystems (23).

Detailed data, sufficient to demonstrate the epidemiology of different MTBC species in different host populations, are difficult to obtain. The epidemiology of the infection differs regionally as well as within different populations in the same region (8, 12, 24).

A crucial aspect of epidemiological data collection is case definition. A case of human tuberculosis may be a patient with a smear-positive sputum sample, a tuberculin reactor, a patient from whom *M. tuberculosis* has been isolated, or a patient with samples positive for any bacteria within the MTBC. Similarly, animal cases may be defined as tuberculin reactors, animals with visible lesions *post mortem*, animals with histological lesions indicative of tuberculosis, or individuals with samples yielding *M. bovis* (or MTBC) in culture. Furthermore, the tuberculin test in animals may be performed as a single or comparative test in the skin of the neck or in the caudal fold, and the interpretation may be different in different situations (e.g., regarding all inconclusive reactions as positive, if a higher test sensitivity is prioritized above specificity) (25). Different case definitions may be used for different purposes, and this must be taken into account when drawing conclusions from data.

## Other Aspects Affecting Prevalence Figures

Isolation of bacteria from each case would enable species identification and molecular typing to support epidemiological investigations. This is, however, not always feasible, and zoonotic transmission (in either direction) of tuberculosis may be overlooked.

In most regions of the world where the prevalence of tuberculosis in animals and humans is high, lack of resources means that diagnostics are insufficient for bacterial identification to species level. Even in high-income countries, diagnostics may rely on microscopy of sputum smears, histological examination of formalin-fixed tissue, culture on media not suitable for all MTBC species, or the use of DNA probes unable to distinguish between MTBC species (7, 13, 22). For example, the addition of glycerol in culture media will enhance growth of *M. tuberculosis* but inhibit growth of *M. bovis*, while culture media for *M. bovis* should preferably include pyruvate (4, 13, 22). Most diagnostic methods require some presumption about expected findings, and this will affect disease monitoring across different populations and geographical regions.

In the absence of bacteriological results, the clinical presentation of tuberculosis cases is sometimes used as an indicator of the causative agent. The primary manifestation of *M. bovis* in humans is usually extrapulmonary, but the proportion of pulmonary cases appears to be increasing (12, 26). Occupational exposure among farmers and abattoir workers usually leads to aerosol infection, this type of exposure may be more frequent in low-income countries with a high prevalence of tuberculosis in animals but where detailed data are scarce (8, 10, 16, 22, 26, 27). Site of infection is, therefore, not a solid basis for conclusions about the causative agent.

## What is in a Name?

Terminology affects risk perception and behavior (28), a fact that is well known in the commercial world but not as commonly discussed in the scientific arena (29). The use of terms like bovine and human tuberculosis would be better applied to mean infection with MTBC in the respective host species. While acknowledging the difference between the species within MTBC and the need to identify each one in each case, it is important not to let the definition of the disease lead to too narrow a perspective, with possible lapses in tracing and control of the infection. Despite public awareness campaigns to combat human tuberculosis, knowledge of the risk presented by infected animals and vice versa can be poor (30), perhaps reflecting a failure in communication and or/awareness among the medical and veterinary professions. The use of terms like “human tuberculosis” and “zoonotic tuberculosis” (2, 3, 26) as contrasting events is not helping disease control, although it may be seen as formally correct.

## Public Health and Animal Disease Control

The primary reason for tuberculosis control in animals is to protect public health. As cattle infected with members of the MTBC can shed the bacteria in milk, unpasteurized dairy products represent a public health risk. In many parts of the world, milk is heat-treated, but other dairy products may be made with raw milk. While pasteurization of all dairy products is crucial, tuberculosis control in cattle and other animals remain important for human and animal health (1, 10, 12, 22, 24). However, all stakeholders may not support disease control efforts, which are perceived as too costly and cumbersome, and therefore, not justifiable, when milk is pasteurized anyway (31). There may be a risk that the progress of tuberculosis control in animals, in combination with pasteurization of milk, has made professionals in human and veterinary medicine less aware, or even ignorant, of the fact that tuberculosis does not restrict itself to one host population. A stronger collaboration between veterinary and public health is often called for, and this call for collaboration must be repeated until taken for granted, and fully implemented in surveillance and control activities.

Many of the most heavily infected regions in the world lack the resources and infrastructure needed to control tuberculosis in animals and humans, while the epidemiology may be entirely different from what has been seen in countries where the disease has been eradicated from the animal population (10, 12, 24). This presents new challenges.

## Conclusion

A systematic collaboration between different professions and control systems, and a common nomenclature clearly separating the name of the host species from the infecting bacterial species are the first steps toward effective tuberculosis surveillance and control.

We should learn from history and not repeat the mistake of those who, upon Koch’s discovery of different MTBC species, first assumed that bovine tuberculosis did not affect humans, leading to the pasteurization of milk for calves long before it was applied to milk for human consumption. The more careful attitude of some early scientists, proposing to omit the host designation of the different bacterial species in order to anticipate assumptions that they are limited to the host whose name they bear (32), should be remembered as we make scientific progress. Understanding the complex ecosystem of the multi-host pathogens within the MTBC is necessary for disease control. There is an urgent need for scientific advances in order to meet the need for global tuberculosis control in all host populations, but this will require a new thinking as regards the ecology of tuberculosis and a practical application of the One Health concept.

## Conflict of Interest Statement

The author declares that the research was conducted in the absence of any commercial or financial relationships that could be construed as a potential conflict of interest.
